# Increased T- and B-cells associated with the phenotype of autoimmune limbic encephalitis with mainly memory dysfunction

**DOI:** 10.1016/j.jtauto.2022.100167

**Published:** 2022-09-28

**Authors:** Niels Hansen, Guido Widman, Demet Önder, Kerstin Schwing, Pitshaporn Leelaarporn, Indra Prusseit, Randi von Wrede, Rainer Surges, Albert J. Becker, Juri-Alexander Witt, Christian E. Elger, Christoph Helmstaedter

**Affiliations:** a Department of Epileptology, University Hospital Bonn, Venusberg - Campus 1, 53127, Bonn, Germany; b Department of Neuropathology, University of Bonn Medical Center, Venusberg - Campus 1, 53127, Bonn, Germany; c Center for Rare Diseases Bonn (ZSEB), University of Bonn, Germany; d Department of Psychiatry and Psychotherapy, Von-Siebold- Str. 5, University of Göttingen, 37075, Göttingen, Germany

**Keywords:** Autoimmune encephalitis, Immune cells, Phenotype, Neuropsychology, Autoantibodies, Verbal memory

## Abstract

**Background:**

Our goal is to investigate the autoantibodies’ presence and immune cells in the bioprobes of autoimmune encephalitis (AE) patients with distinct phenotypes as a promising target in AE.

**Methods:**

We retrospectively analyzed immune cells via flow cytometry, serum and cerebrospinal fluid (CSF) autoantibodies, electroencephalography, magnetic resonance imaging in 94 AE patients with suspected temporal lobe epilepsy and classified neuropsychological phenotypes according to their occurrence.

**Results:**

We detected different phenotypes in 94 AE patients [10.6% with isolated memory dysfunction (MEM), 11.7% with mood-dysfunction, 12.7% with mood and memory dysfunction, 13.8% with memory and attention dysfunction, 18.1% with memory, mood and attention disturbances and 20.2% with no mood, memory or attention dysfunction]. We did discern a relevant association of phenotypes and CSF antibody-positivity on CSF CD4^+^ T-cells, CD8+T-cells and HLADR + CD8+T-cells in our patients with MEM presenting elevated CD8+T-cells and HLADR + CD8+T-cells. Furthermore, CSF CD19+B-cells differed significantly between phenotypes in patients with MEM.

**Discussion:**

Taken together, the phenotypes in combination with CSF antibody-positivity are biomarkers for stratifying patients. Furthermore, our results confirm the role of CD4^+^ T-cells, CD8+T-cells and CD19+B-cells in AE patients with a memory dysfunction, providing insights into AE pathogenesis. Our preliminary results should be confirmed by larger-scale investigations.

## 1 Introduction

Immune-cell subsets are interesting candidates for advancing the diagnosis and treatment of autoimmune encephalitis (AE) - a dynamic disease comprising different clinical features ranging from seizures to cognitive, memory, mood alterations or psychosis [[1], [2], [3]]. The underlying pathomechanism involved in most clinical features is unknown. Although very seldom, specific clinic phenomena such as faciobrachial dystonic seizures might suggest the underlying pathomechanism of encephalitis, such as LGI1-antibodies in AE with faciobrachial dystonic seizures [4]. Such specific clinical features are important to enable a rapid therapeutic intervention to prevent further cognitive deterioration [5]. However, as neuropsychiatric features often overlap in patients, it is difficult to differentiate clinical syndromes and plan treatment strategies. It is therefore highly relevant to have additional biomarkers that can be used to identify patients for early immunotherapy. Flow cytometry is an interesting tool for investigating immune cells as biomarkers, as it is easy to perform and has delivered promising results, especially regarding its clinical applicability in patients with neuropsychiatric disorders [[6], [7], [8], [9], [10], [11]]. We thus explored the usefulness of flow cytometry to immunophenotype patients with distinct neuropsychological phenotypes of AE. Furthermore, we aim to explore the significance of the presence of serum neural and intracellular autoantibodies for cognitive and mood functions in AE phenotypes.

## 2 Methods

In this retrospective, observational study we included 94 patients with possible and definitive AE according to the Graus criteria [1] and suspected temporal lobe epilepsy suggesting limbic encephalitis and without any immunotherapy ≤3 month prior to flow cytometry. They underwent flow cytometry in the Department of Epileptology, University of Bonn. Patients were classified as “antibody-positive” if actual antibodies were detected in the CSF or PB. Previous detection of antibodies and/or antibody proof at the detection limit in patients were categorized as “antibody-negative” patients. Furthermore, thyrosine peroxidase (TPO) or antinuclear antibodies (ANA) as the only presenting antibodies were categorized as “antibody-negative” patients, as these autoantibodies might argue for an underlying autoimmune disorder other than autoimmune encephalitis, such as autoimmune thyroiditis. The presence of additional unknown bands in western blot, as well as cytoplasmatic immunoreactivity in cerebellar and hippocampal rat brain sections after incubation with the patient's serum were further characterized as “antibody-positive” patients. Specific antibodies were detected in the neuropathology laboratory at the University of Bonn via immunoblots [paraneoplastic antibodies: Amphiphysin, collapsing response mediator protein 5 (CRMP5)/cronveinten 2 (CV2), Hu, Ma-2/Ta, Recoverin, Ri, SOX1, Titin and Yo] and cell-based assays [Aquaporin 4 receptors, α-amino-3-hydroxy-5-methyl-4-isoxazolepropionic acid receptor (AMPAR) 1, AMPAR2, contactin-associated protein 2 (CASPR2), Gamma-aminobutyric acid A/B (GABAA/B) receptors, glutamic acid decarboxylase 65/67 (GAD65/67), leucine-rich glioma inactivated 1 (LGI1) and *N*-methyl-d-aspartate (NMDA) receptor]. All patients underwent electroencephalography (EEG), magnetic resonance imaging (MRI) and neuropsychological assessment (see section neuropsychological assessment). A 3 T MRI was used to conduct neuroimaging of the brain at the Life and Brain Institute (Magnetom Trio, Siemens, Germany) and/or in the Department of Neuroradiology (Philips Medical Systems, Germany), University of Bonn. To assess signal changes in the temporal lobe typical for encephalitis, we applied a sum score previously described in more details [[6], [7]]. We employed these specifications: 1 = unilateral hippocampal or amygdalar signal or volume increase, unilateral blurring of the interior hippocampus part or unilateral volume decrease in hippocampus, 2 = bilateral volume increase in hippocampal or amygdalar volume or bilaterally-blurred interior hippocampus or bilaterally-decreased volume of the hippocampus. All patients underwent EEG (System Plus evolution, Micromed S. p.A, Treviso, Italy) to diagnose epilepsy and AE. An EEG criterion for AE according to Graus [1] was fulfilled if epileptic potentials or slow waves were observed in the temporal lobe. To score CSF parameters, we applied these specifications relying on laboratory records from the Department of Clinical Chemistry and Clinical Pharmacology, University of Bonn: the existence of a blood-brain barrier dysfunction was rated according to these specifications based on the albumin-quotient: 0 = no blood-brain barrier disturbance and 1 = blood-brain barrier disturbance. In addition, the presence or absence of intrathecal immunoglobulin (IgG) synthesis in the CSF relies on the Reiber formula [12] with this classification system: 0 = absence of intrathecal immunoglobulin (IgG) synthesis and 1 = presence of intrathecal IgG synthesis. The presence of intrathecal IgG synthesis is attributable to the presence of oligoclonal bands, which were evaluated in the Department of Clinical Chemistry and Clinical Pharmacology at the University of Bonn via isoelectric focusing and an electrophoresis system. The presence of isolated oligoclonal bands in cerebrospinal fluid was considered pathological. Lack of oligoclonal bands or the conjunction of oligoclonal CSF ligaments as well as serum oligoclonal ligaments were considered non-pathological. All these CSF investigations were conducted by employees in the Department of Clinical Chemistry and Clinical Pharmacology, University of Bonn. All patients agreed to these clinical procedures via informed consent before the investigations. Our study concurred with the Declaration of Helsiniki and was approved by our local ethics committee in the Medical Faculty of the University of Bonn.

### 2.1 Neuropsychological assessment

Every patient underwent a test battery examining verbal, figural memory and attentional-executive function. We used the revised version “Diagnosticum for Cerebralschädigung” (DCS-R) to measure figural memory capacity [13] and to assess verbal memory function, we utilized the “Verbaler Lern-und Merkfähigkeitstest” (VLMT) [14]. Each patient's neuropsychological performance was scored on a numerical rating scale in relation to standard performance - meaning that the performance is classified as 0 = if lower than 3-fold below the standard deviation of the mean, 1 = if two-fold below the standard deviation of the mean, 2 = if 1-fold below the standard deviation of the mean, 3 = if within ±1 standard deviation of the mean and 4 = if one-fold above the standard deviation of the mean.

### 2.2 Phenotype classification

Patients were subdivided according to these neuropsychological and clinical assessments into four categories: (1) memory impairment affecting verbal and/or figural capacity (score ≤2) (MEM), (2) impaired attentional-executive function with a score ≤2 (ATT), and (3) evidence of mood dysfunction in patient history (score = 1 means mood dysfunction, score = 0 no mood dysfunction) (PSY). We further categorized phenotypes due to their occurrence. We considered only phenotypes with patient numbers ≥10 as relevant. The mixed phenotype affecting mood and attentional functions (PSY + ATT) only appeared in three patients and was thus not further considered a relevant phenotype. No patients presented the pure phenotype ATT. In addition, mixed phenotypes affecting memory and mood (MEM + PSY), memory, attention and mood (MEM + PSY + ATT) were selected. We observed patients who revealed no dysfunctionality in memory, mood and attention measures (MEM-ATT-PSY-).

### 2.3 Immune cells

We assessed immune cells in CSF and PB via flow cytometry using a BD LSR Fortessa flow cytometer (BD Bioscience, California, USA). One investigator blinded to the patients analyzed the flow cytometry with the gating strategy of leukocytes using Kaluza software (Beckman Coulter GmbH, Life Science, Krefeld, Germany). The gating strategy we applied to differentiate T- and B-cell subsets has been described via commonly used cell-subset markers [10]. CSF samples were obtained via lumbar punctures that were then processed via polypropylenes tubes. Blood samples were put into EDTA monovettes. Cells were separated from CSF by centrifugation steps (first: 290 g, 15 min, 4°; second/third: 290 g, 15 min, 21°). In addition, Versa Lyse buffer (Beckman Coulter, Germany) was used to segregate cells from CSF and blood. In this study, we examined two important immune cell populations: T lymphocytes (T-cells) and B lymphocytes (B-cells). These immune cells were grouped according to their differentiation cluster (CD) as well as cellular surface receptors and major histocompatibility class II into human leukocyte antigen DR isotypes (HLA-DR+) CD4^+^ T cells, HLA-DR + CD8^+^ T cells, CD138+ B cells and CD19^+^ B cells from blood and CSF. The CSF HLA-DR+/CD8+ T-cells represent only the activated CD8^+^ T-cells, whereas the CSF HLA-DR+/CD4+ T-cells depict only the activated CD4^+^ T-cells. For immune cell specification, we referred to these fluorochromo-conjugated antibodies in T- and B-lymphocyte populations: (Beckman-Coulter) CD19-Alexafluor700, CD138-PE, HLA-DR-ECD, CD4-APC and 700CD8-PacificBlue. As a gating strategy for selecting blood and CSF leukocyte subpopulations, we applied mainstream cell line markers [10]. Our main focus was on B- and T-cell populations, which likely play a relevant role in limbic encephalitis according to published evidence [8,9]. We analyzed the following immune cells in PB and CSF applying a formula in the manufacturer's recommendations [CD19^+^ B-cells, CD138+ B-cells, CD4^+^ T-cells, human leukocyte antigen DR isotype (HLA-DR+) CD4^+^ T-cells, CD8^+^ T-cells and HLA-DR + CD8^+^ T-cells as well as CD4/8+ T-cell ratio in PB and CSF]. We determined absolute cell numbers following the manufacturer's guidelines in the Kaluza software instructions of Beckman Coulter GmbH.

### 2.4 Statistics

Statistical analysis was done via Sigma Statistics (Version 11, 2008, San Jose, California, USA). In addition figures were constructed by CorelDraw (Graphics Suite Version 11, Ontario, Canada). Two two-way ANOVAs with (1) phenotype and (2) autoantibody positivity in PB as factors as well as (1) phenotype and (2) autoantibody positivity in CSF as factors served to evaluate differences between immune cells and other laboratory parameters such as oligoclonal bands and a blood-brain barrier disturbance in the CSF, MRI scores, and in EEG scores. The receiver operating characteristic curves (ROC) analyses were performed using the software Excel Analyse-it. A p-level of <0.05 was considered as significant.

## 3 Results

### 3.1 Phenotyping of patients

We investigated 94 patients aged on average 43 ± 15 years with possible und definitive AE and suspected temporal lobe epilepsy (Table 1). We detected these clinical phenotypes: 10 of 94 (10.6%) patients with MEM, 11 of 94 patients (11.7%) with PSY, 12 of 94 patients (12.7%) with MEM + PSY, 13 of 94 patients (13.8%) with MEM + ATT, 17 of 94 patients (18.1%) with MEM + PSY + ATT and finally 19 of 94 patients (20.2%) MEM-PSY-ATT-.

**Table 1:** Laboratory parameter of autoimmune encephalitis phenotypes.

PARAMETER	PHENOTYPES						
	MEM- PSY- ATT-	MEM	MEM + PSY	MEM + ATT	MEM + PSY + ATT	PSY	ANOVA
N	19	10	12	13	17	11	
Age at flow cytometry (y)	41 ± 14	47 ± 17	39.6 ± 15	48.2 ± 16	41.6 ± 12.5	40.3 ± 11.8	ns
Gender, female, N (%)	11 (59%)	2 (20%)	4/12 (33%)	5/13 (38%)	8 (47%)	5 (45%)	ns
CSF intrathecal IgG synthesis (%)	5 (%)	2 (20%)	3/12 (25%)	4/13 (31%)	8 (47%)	1 (9%)	ns
CSF BBB disturbance N (%)	3 (%)	4 (40%)	2/12 (16.6%)	0/13 (0%)	4 (24%)	0 (0%)	ns
CSF CD19^+^ B-cells (cells/ml)	5 ± 1.17	181 ± 169	5.4 ± 2.2	112 ± 99	10 ± 4.05	4.1 ± 1.76	#
CSF CD138+ B-cells (cells/ml)	0.2 ± 0.08	50 ± 46	0.17 ± 0.09	2.9 ± 2.6	2 ± 1.74	0.07 ± 0.06	ns
CSF CD4^+^ T-cells (cells/ml)	419 ± 149	2015 ± 1707	333 ± 156	863 ± 504	530 ± 155	422 ± 146	+
CSF HLA-DR + CD4^+^ T-cells (cells/ml)	74 ± 35	366 ± 319	76 ± 39	124 ± 54	161 ± 58.3	109 ± 52	ns
CSF CD8^+^ T-cells (cells/ml)	78 ± 31	785 ± 683	92 ± 43	120 ± 39	106 ± 28.7	115 ± 37	*
CSF HLA-DR + CD8^+^ T-cells (cells/ml)	41 ± 12+	488 ± 431+	46 ± 21+	57 ± 19+	71 ± 23+	80 ± 29+	*
CSF CD4/8+ T-cells (cells/ml)	2.4 ± 0.55	4.59 ± 1.07	3.79 ± 0.47	5.5 ± 10.4	5.7 ± 1.5	5 ± 1.08	ns
Blood CD19^+^ B-cells (cells/ml)	139,088 ± 31,066	122,332 ± 40,123	144,081 ± 40,581	166,704 ± 42,226	217,467 ± 111,329	118,317 ± 31,424	ns
Blood CD138+ B-cells (cells/ml)	1541 ± 725	22,146 ± 20,269	617 ± 257	3640 ± 1896	2571 ± 1519	874 ± 299	ns
Blood CD4^+^ T-cells (cells/ml)	658,738 ± 129,022	378,388 ± 101,358	565,417 ± 133,429	689,650 ± 180,367	1,119,482 ± 525,249	501,683 ± 126,633	ns
Blood HLA-DR + CD4^+^ T-cells (cells/ml)	31,347 ± 6329	22,345 ± 6411	29,707 ± 12,824	70,939 ± 30,126	72,584 ± 35,403	26,445 ± 6650	ns
Blood CD8^+^ T-cells (cells/ml)	226,171 ± 48,933	201,179 ± 68,745	262,891 ± 116,597	222,782 ± 52,249	396,251 ± 207,454	193,980 ± 43,317	ns
Blood HLA-DR + CD8^+^ T-cells (cells/ml)	23,787 ± 6871	34,098 ± 9868	19,341 ± 4952	57,164 ± 23,331	47,658 ± 19,611	26,964 ± 6450	ns
Blood CD4/8+ T-cells (cells/ml)	3 ± 0.35	3.03 ± 1.01	3.3 ± 0.68	3.66 ± 0.82	4 ± 0.83	3 ± 0.46	ns
MRI score (0–12)	2.19 ± 0.5	3.3 ± 0.36	2.1 ± 0.53	1.8 ± 0.31	2.2 ± 0.5	2 ± 0.69	ns
EEG score (0–6)	2.8 ± 0.51	3.4 ± 0.42	3.8 ± 0.39	2.7 ± 0.61	2.9 ± 0.51	3.4 ± 0.55	ns

**Abbreviations:** BBB = blood brain barrier, CSF = cerebrospinal fluid, EEG = electroencephalography, HLA-DR = human leukocyte antigen – DR isotype, IgG = immunoglobulin G, MRI = magnetic resonance imaging, ns = non-significant, y = years. *ANOVA with factor phenotype, CSF antibody positivity and interaction between factors; p < 0.05, #p ANOVA with factor phenotype, p < 0.005. + ANOVA with factor phenotype, and interaction between factors, p < 0.005.

### 3.2 Neural autoantibodies in patients

The AE patients comprised 29/94 (31%) antibody-positive patients (n = 5 GAD65 PB + CSF, n = 2 GAD65 PB, n = 1 GAD65 CSF, n = 1 CASPR2 PB + CSF, n = 1 CASPR2 CSF, n = 2 NMDAR PB, n = 2 Recoverin PB, n = 1 Zic4 PB, n = 1 Titin PB, n = 1 Yo PB, n = 1 LG1 PB, n = 1 CV2, n = 1 Ri CSF, n = 2 additional bands in western blot CSF + PB, n = 4 additional bands in western blot, unspecific neuronal binding pattern in rat brain sections n = 2 PB, n = 1 cytoplasmatic binding pattern in rat brain sections CSF + PB), and 65/94 (69%) antibody-negative patients.

### 3.3 Association of phenotypes, serum and CSF antibody positivity with immune cells

**Fig. 1:**
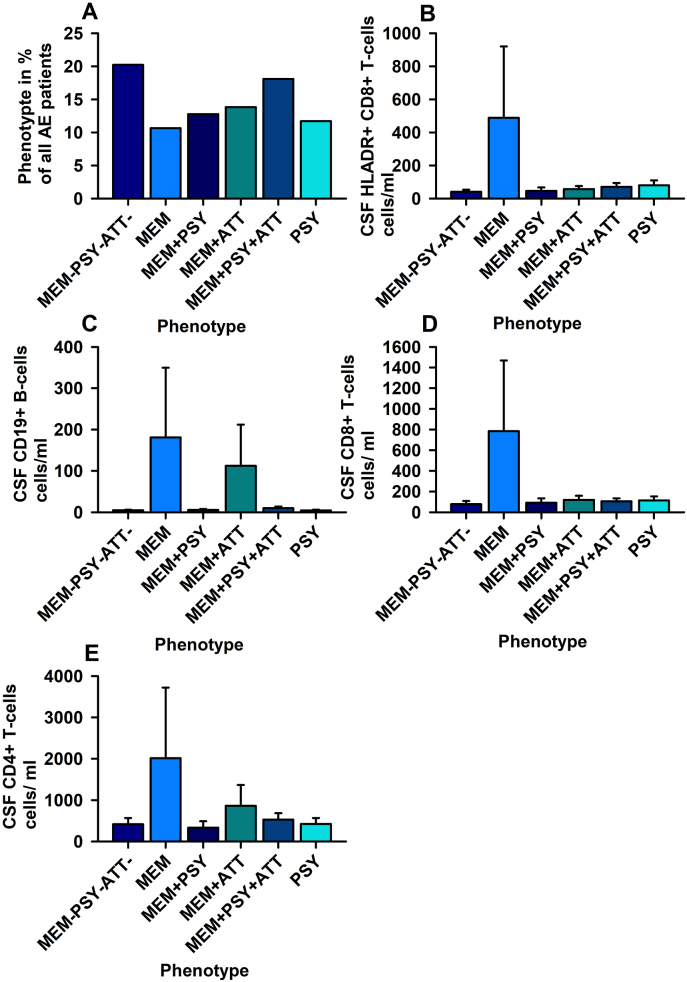
Elevated CD4^+^ T-cells, CD8+T cells and CD19^+^ B-cells are associated with the phenotype of autoimmune encephalitis with mainly memory impairment. In A the distribution of phenotypes among our cohort of patients with autoimmune limbic encephalitis is shown. In B elevated HLADR + CD8^+^ T-cells, in C ascended CD19^+^ B-cells, in D increased CD8^+^ T-cells and in E elevated CD4^+^ T-cells are shown in MEM phenotype. The phenotype is a relevant factor determining the differences between HLADR + CD8^+^ T-cells in B, CD19^+^ B-cells in C, CD8^+^ T-cells in D and CD4^+^ T-cells in E among phenotypes. *p < 0.005 two factorial ANOVA. Abbreviations: MEM = phenotype with memory dysfunction, PSY = phenotype with mood dysfunction, MEM + PSY = phenotype with mood and memory dysfunction, MEM + ATT = phenotype of memory and attentional dysfunction, MEM + PSY + ATT = phenotype of memory, attentional-executive and mood dysfunction, MEM-PSY-ATT- = phenotype without affection of memory, mood and attentional-executive functions.

The phenotypes and CSF and serum antibody-positivity factors demonstrate no relevant association with immune cells in the PB (CD19^+^ B-cells, CD138+ B-cells, CD4^+^ T-cells, HLA-DR + CD4^+^ T-cells, CD8^+^ T-cells, HLA-DR + CD8^+^ T-cells and CD4/8+ T-cell ratio; data not shown). However, the phenotype and CSF serum antibody positivity factors showed a relevant association with CSF CD8^+^ and CD4+T-cell differences with a rise in CSF CD8^+^ T-cells (Factor phenotype: CD8^+^ T-cells: ANOVA F = 8.2, p < 0.001; factor CSF antibody positivity: F = 5.3, p < 0.05; interaction between phenotype and CSF antibody positivity: F = 8.6, p < 0.001; Fig. 1), CD4^+^ T-cells (Factor phenotype: CD8^+^ T-cells: ANOVA F = 5.7, p < 0.001; factor CSF antibody positivity: ns; interaction between phenotype and CSF antibody positivity: F = 7.0, p < 0.001; Fig. 1), and HLADR + CD8^+^ T-cell differences with increased HLADR + CD8^+^ T-cells (HLADR + CD8^+^ T-cells: Factor phenotype, ANOVA: F = 8.2, p < 0.001; factor CSF antibody positivity, ANOVA: F = 5.3, p < 0.05; interaction between these factors, ANOVA: F = 8.7, p < 0.001; Fig. 1), but not on other immune cell subsets in CSF (CD138+ B-cells, CD4^+^ T-cells HLA-DR + CD4^+^ T-cells and CD4/8+ T-cell ratio). However, post hoc testing revealed no relevant differences in CSF CD4^+^ T-cells, CD8^+^ T-cells, HLADR + CD8^+^ T-cells and HLADR + CD4^+^ T-cells between clinical phenotypes. The phenotype is also a relevant factor for differentiating CD19^+^ B-cells in patients (ANOVA: F = 4, p < 0.005) with the MEM and MEM + ATT phenotype showing a rise in CD19^+^ B-cells (Fig. 1). In addition, post hoc testing revealed no relevant differences in CSF CD19^+^ B-cells between clinical phenotypes. The serum antibody-positivity factor had no relevant association with CSF immune cells (CD19^+^ B-cells, CD138+ B-cells, CD4^+^ T-cells, LA-DR + CD4^+^ T-cells, CD8^+^ T-cells, HLA-DR + CD8^+^ T-cells and CD4+/8+ T-cell ratio).

Furthermore, we determined optimized thresholds of CD19^+^ B-cells, CD4^+^ T-cells, CD8^+^ T-cells and HLADR + CD8^+^ T-cells to distinguish between unaffected patient (MEM-ATT-PSY- phenotype) and affected patient phenotypes (all other phenotypes pooled together) [CD19+B-cells: AUC = 0.51, p = 0.90, optimal threshold: 0.35 cells/ml, TPF (sensitivity) = 0.84, FPF (1- specificity) = 0.77; CD4+T-cells: AUC = 0.54, p = 0.62, optimal threshold: 65.42 cells/ml, TPF = 0.84, FPF = 0.63; CD8+T-cells: AUC = 0.51, p = 0.86, optimal threshold: 6.06 cells/ml, TPF = 0.95, FPF = 0.86; HLADR + CD8^+^ T-cells: AUC = 0.50, p = 0.95, optimal threshold: 223.39 cells/ml, TPF = 1, FPF = 0.89] as well as those patients with the MEM + phenotype and all other phenotypes [CD19+B-cells: AUC = 0.55, p = 0.70, optimal threshold: 0.66 cells/ml, TPF = 0.78, FPF = 0.50; CD4+T-cells: AUC = 0.55, p = 0.68, optimal threshold: 1376 cells/ml, TPF = 0.93, FPF = 0.80; CD8+T-cells: AUC = 0.531, p = 0.764, optimal threshold: 0.86 cells/ml, TPF = 0.99, FPF = 1; HLADR + CD8^+^ T-cells: AUC = 0.52, p = 0.86, threshold: 0 cells/ml, TPF = 0.99, FPF = 1]. In addition, we calculated optimized thresholds of CD19^+^ B-cells, CD4^+^ T-cells, CD8^+^ T-cells and HLADR + CD8^+^ T-cells to distinguish between patients with blood-brain barrier dysfunction and those without a blood -brain barrier dysfunction [CD19+B-cells: AUC = 0.55, p = 0.62, optimal threshold: 35.35 cells/ml, TPF = 0.96, FPF = 0.85; CD4+T-cells: AUC = 0.55, p = 0.55, optimal threshold: 3.07 cells/ml, TPF = 1, FPF = 0.33; CD8+T-cells: AUC = 0.52, p = 0.85, optimal threshold: 7265.24 cells/ml, TPF = 1, FPF = 0.92; HLADR + CD8^+^ T-cells: AUC = 0.53, p = 0.67, threshold: 322.96 cells/ml, TPF = 1, FPF = 0.85)]. These results fail to support these biomarkers in differentiating between clinical phenotypes.

### 3.4 Association with phenotypes, serum and CSF antibody positivity on CSF, EEG and MRI parameter

Serum autoantibody positivity was a relevant factor for the presence of oligoclonal bands (ANOVA: F = 11.3, p < 0.05). Furthermore, the phenotype had an relevant association with blood brain barrier disturbance (ANOVA F = 2.7, p < 0.05). Phenotypes, serum, and CSF antibody positivity have no relevant influence on EEG and MRI scores.

## 4 Discussion

Our main findings suggest that CD8^+^ T-cell subsets in CSF serve as biomarkers to distinguish AE's neuropsychological phenotypes. The phenotype primarily characterized by isolated memory impairment is associated with more CSF CD8^+^ T-cells. Elevated CD8^+^ T-cells in CSF in patients with pure memory dysfunction is further corroborated by autoantibody positivity in CSF. A relevant interaction between CSF autoantibody positivity and phenotypes in elevated CD8^+^ indicates that both autoantibodies and CD8^+^ T-cells might contribute to the primarily memory impairment phenotype in AE. Nevertheless, CSF autoantibody positivity's contribution must be interpreted with caution, as we had too few CSF autoantibody-positive patients to draw robust conclusions. The CD8^+^ T-cells thus are helpful to characterize the memory impairment phenotype in comparison with other phenotypes that are probably accompanied by a relevant T-cell immunopathology to stratify patients more accurately for immunotherapeutic approaches. The relevant role of CD8^+^ T-cells in the pathogenesis of autoimmune limbic encephalitis was recently shown for anti-GAD65 limbic encephalitis [15], limbic encephalitis in temporal lobe epilepsy patients [16] and GABA-B receptor limbic encephalitis [17], thus confirming our findings. However, no study so far has addressed the exclusive role of activated CD8^+^ T-cells in a phenotype of limbic encephalitis involving prominent memory disturbances behind the CD8^+^ T-cell driven pathophysiology of memory dysfunction in limbic circuits. Our findings also suggest that the phenotype of a pure memory impairment or demarcation of other phenotypes might give us some hints about AE's underlying pathophysiology. On the functional level, our findings do reveal the presence of CSF CD19^+^ B-cells that play a crucial role in producing autoantibodies and are associated with impaired memory performance in AE patients. Moreover, and in line with these observations, is the recent evidence that CD19^+^ B-cells as antibody-producing cells play a role in figural memory performance [18]. The key role of autoantibodies in verbal memory dysfunction might be related to (1) the known deposition of autoantibodies in the human hippocampus in autoimmune encephalitis with limbic features known from neuroimaging studies [19,20] after a postulated transient breakdown of the blood-brain barrier, and (2) the human hippocampus' crucial role in verbal memory formation [[21], [22], [23]]. Other functions such as attention, global cognition, or mood often involve temporal and extratemporal brain networks that might be dysfunctional in AE [24], but they are less often affected by immunoglobulin depositions in various AE-associated antibody subtypes. We postulate a temporal location of immunoglobulin depositions in our patients with suspected temporal lobe epilepsy due to antibody-positive AE. The prominent role of human neuronal autoantibodies in memory performance associated with AE has been confirmed in murine passive transfer models of AE from men to mice, revealing that human cerebrospinal fluid NMDA receptor antibodies induce AE [25] affecting memory performance [26,27] by disrupting NMDA receptor synaptic function. There is indirect evidence from antibody-mediated memory-dysfunction research in humans, as memory disturbances in NMDA receptor encephalitis patients were reversed by depleting B-cells via rituximab [28]. Together with those studies, our findings highlight the important role autoantibodies and CD19^+^ B-cells play in disease-related memory dysfunction - probably due to structural changes in the temporal lobe [29]. Several autoantibodies which were also present in our phenotype subgroups, such as GAD65-, LGI1-, and NMDA receptor-antibodies are known to be associated with verbal memory decline [29,30] in patients with AE, although each antibody exhibits its own mechanism, ie, synaptic-receptor dysfunction in the case of NMDA receptors or LGI1-antibodies [25,26], or altered synaptic transmission via presynaptic alterations in GABA release due to GAD65-antibodies acting in concert with additional antibodies [31]. Despite subordinate mechanisms in autoantibody-mediated encephalitis, the crucial role antibodies and CD19^+^ B-cells play in memory dysfunction with therapy implications must be kept in mind when treating patients with predominant or pure memory disturbances.

### 4.1 Limitations

As patients often display impairment in various functional aspects such as mood, cognition, and memory [3], clinical phenotypes often cannot be strictly differentiated from each other. However, careful observation is necessary to differentiate a purely clinical phenotype with a memory or psychiatric manifestation. Often clinical phenotypes show a substantial overlap between neuropsychological subdomains such as cognition, memory, and mood functions, as recently shown in conjunction with NMDA-receptor encephalitis [32], so that our investigation has to be proven in more large cohort studies to be of practical feasibility. Another critical issue is our small cohort of heterogeneous subgroups with serum and even less CSF antibodies, limiting our findings’ significance. Another limitation is that we could not draw clinically relevant conclusions related to the question of cerebrospinal fluid autoantibodies associated with memory function due to too small samples of CSF autoantibodies. Furthermore, it would make sense to investigate these T- and B-cell subsets in limbic encephalitis with memory dysfunction in comparison to control subjects. Another aspect to carefully consider is that the level of CD8^+^ T-cells and CD19^+^ B-cell expression might change as the disease develops. This might explain why our results reveal discrepancies in B-cell subset populations (CD19^+^ vs. CD138+ B-cells). This point should be kept in mind taking a longitudinal approach in future research. In addition, note that it would be of great interest to investigate kappa-free light chains in conjunction with oligoclonal bands in a future study to better distinguish non-inflammatory and inflammatory diseases, and to evaluate intrathecal IgG synthesis, as recently illustrated in a study by Konen et al. [33].

### 4.2 Conclusions

Taken together, our study reveals that CD8^+^ T-cells and CD19^+^ B-cells might play a relevant role as an additional biomarker by which (1) to differentiate the often overlapping neuropsychological phenomenology of AE patients and (2) further to stratify patients for immunotherapy. However, the relevance of the biomarkers of CD4^+^ T-cells, CD8^+^ T-cells and activated CD8^+^ T-cells is not supported by our ROC analysis. Thus, these cells might play key roles in the pathophysiology of AE, although these biomarkers do not seem suitable via the current assessment strategy for distinguishing clinical phenotypes, including those associated with memory dysfunction. Novel techniques should be developed to exploit the potential of standard flow cytometry. Such an enriched flow cytometry technique would include the potential assessment of the flow-cytometric functional immune phenotyping matrix as described in the literature [34,35] to delineate differences of the immune repertoire between clinical phenotypes. The pure manifestation of memory impairment without affecting other neuropsychological functions are likely associated with elevated CD4^+^ T-cells, CD8^+^ T-cells and CD19^+^ B-cells that might bear clues for the pathogenesis of memory dysfunction in these AE. In addition, for memory impairment in AE the occurrence of CSF autoantibodies and CD8^+^ T-cells seems to be important although conclusions here are limited to the small patient size. We believe that our findings highlight the pathophysiological role of activated CD8^+^ T-cells in deciphering AE phenotypes. Cutting-edge immunodiagnostics including flow cytometry help us to provide more insights into immune cells and their contribution to neuropsychological functions impaired by AE to be addressed in further large-scales studies.

## Credit author statement

**NH and CH** conceptualized the study, **NH and JAW** wrote the manuscript, **DÖ and KS** contributed to data collection, all authors have read and revised for important intellectual content the published version of the manuscript.

## Declaration of competing interest

The authors declare that they have no known competing financial interests or personal relationships that could have appeared to influence the work reported in this paper.

## Data Availability

Data will be made available on request.
